# Detection of bumblebee behaviour around a nest box using AI-based image analysis

**DOI:** 10.1016/j.mex.2026.103822

**Published:** 2026-02-14

**Authors:** Katsumi Ohyama, Hiroki Naito, Norio Hirai

**Affiliations:** a Graduate School of Sustainable System Sciences, Osaka Metropolitan University, 1-1 Gakuencho, Sakai, Osaka 599-8531, Japan; b Graduate School of Agricultural and Life Sciences, The University of Tokyo, 1-1-1 Yayoi, Bunkyo, Tokyo 113-8657, Japan; c Graduate School of Agriculture, Osaka Metropolitan University, 1-1 Gakuencho, Sakai, Osaka 599-8531, Japan

**Keywords:** Algorithm, Industrial camera, Pollinator, Strawberry, YOLO

## Abstract

Insect pollinators, such as bumblebees, are commonly used to facilitate pollination in strawberry greenhouses. To ensure effective pollination, this study monitored the entry and exit behaviour of bumblebees around the nest box using an industrial camera. Video footage was captured, and a virtual cube-shaped frame was positioned at the entrance of the nest box. Bumblebee detection and counting within this virtual frame were performed using two methods: a) YOLO only and b) YOLO with a simple algorithm. In the algorithm-based method, if a bumblebee crossed the virtual frame an odd number of times, it was classified as having entered or exited the nest box. Conversely, an even number of crossings indicated that the bumblebee had either turned back or re-entered without exiting.

The proposed method allowed for the automated counting of bumblebees entering and exiting a nest box.

Compared to the YOLO-only method, the proposed method significantly improved performance metrics, including accuracy, precision, and F1 score.

The proposed method can effectively support the monitoring of pollinator behaviour in strawberry greenhouses.

## Graphical abstract

**Figure:**
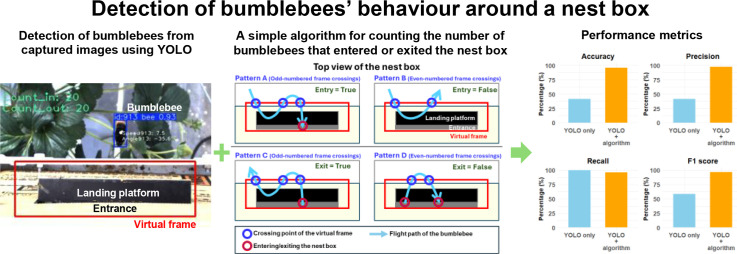

## Specifications table

**Table:** 

**Subject area**	Agricultural and Biological Sciences
**More specific subject area**	Pollinator behaviour
**Name of your method**	Detection of pollinator behaviour using AI-based image analysis
**Name and reference of original method**	N/A
**Resource availability**	N/A

## Background

Most of the strawberry productions in Japan are carried out in greenhouses owing to consumer demand for high-quality fruit [1,2]. Within these greenhouses, pollinators provide essential services to ensure the production of high-quality strawberries [3,4]. Among the pollinators, bumblebees are recognized as one of the most important species [5]. In strawberry production, bumblebees are commonly introduced to improve fruit yield and quality [6,7]. Hence, in strawberry greenhouses, suitable conditions for both the pollinators and the strawberry plants must be established to achieve sustainable production. As strawberry cultivation techniques continue to advance [8], a deeper understanding of bumblebee behaviour is required to ensure efficient pollination service.

In recent years, artificial intelligence (AI) and computer vision have received increasing attention in the field of entomology [9]. Traditionally, bumblebee activity, such as the number of individuals entering or exiting a nest box, has been counted manually via visual observation [10] or video [11]. However, to investigate bumblebee activity more effectively, several technologies, such as the applications of RFID [12,13], radar [[14], [15], [16]], audio signature [17,18], and camera systems [19,20], have been investigated [21]. Despite these progresses, there is a need for a simpler and more accurate system to monitor bumblebee activity and better understand their behaviour.

The aim of this study was to investigate the behaviour of bumblebees in commercial strawberry greenhouses. As an initial step, we developed an automatic method for estimating the number of bumblebees that enter or exit a nest box using AI-based image analysis with a simple algorithm.

## Method details

### Study sites

A commercial strawberry greenhouse located in Miyagi prefecture, Japan (37°55′N, 140°53′E), was used as a model greenhouse. The model greenhouse consisted of five east-west-orientation spans (40 m × 45 m), in which approximately 12,000 strawberry plants (*Fragaria* × *ananassa*) were cultivated from August to June. In the greenhouse, bumblebees (*Bombus ignitus*) were used for pollination. A bumblebee nest box was provided by a vendor (Agrisect Inc., Japan) and replaced periodically with a greenhouse grower (usually every 2-3 months). In the present experiment, the nest box was replaced approximately 1 month before being examined.

### Video image capturing and model development

**Fig. 1:**
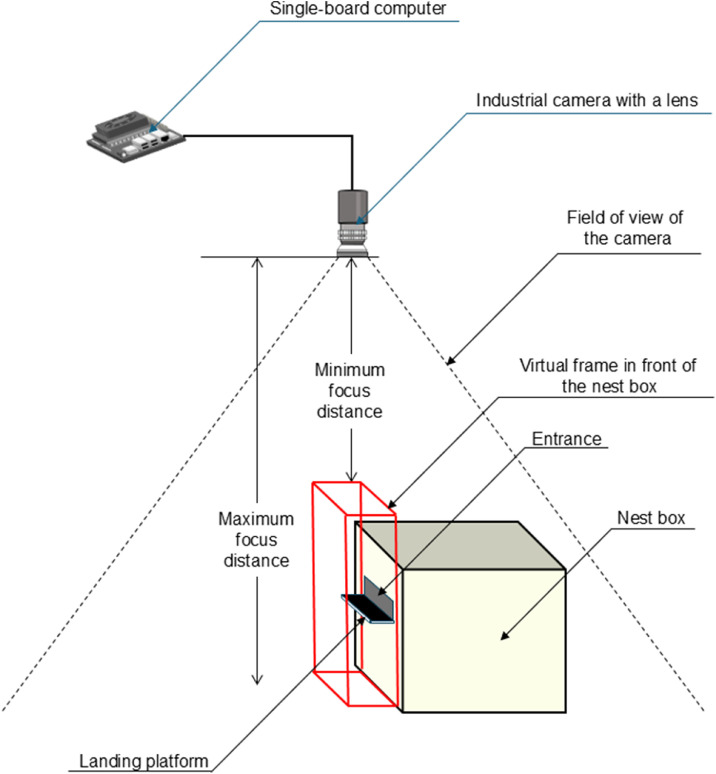
Schematic diagram of the experimental setup and the virtual frame positioned in front of the nest box.

To monitor the bumblebee activities around the entrance of the nest box, an industrial camera (DFK33UX273; The Imaging Source, LLC) with a lens (VS-0620VM; VS Technology Co.) was installed 0.5 m above the nest box (Fig. 1). The camera was connected to and controlled using a single-board computer (Jetson Orin Nano Developer kit, NVIDIA Co.). Video images were recorded at a frame rate of 240 fps. The gain and shutter speeds of the camera were controlled automatically.

In the present experiment, an independent file was created every ten minutes and stored on a single-board computer and in the cloud from 6:00 to 18:00. For model training, still images were extracted from video footage recorded on 6 February 2025 to train a bumblebee detection model using YOLO11x [22]. From the still images, 1000 images were used for training, and 200 images were used for validation to fabricate a trained model with a custom dataset.

### Detection of bumblebees

Before bumblebee detection, an arbitrary cube-shaped virtual frame was placed in front of the entrance to the nest box (Fig. 1). In the present experiment, the cross-sectional size of the virtual frame was set to 495 × 150 pixels in the video images (640 × 480 pixels). Bumblebee detection and entry/exit were performed using a PC (operating system: Windows 11, Python 3.12.3 interpreter). The trained YOLO model was employed to detect the bumblebees.

To count the number of bumblebees that entered and exited the nest box, two AI-based image analysis methods were evaluated:

1) YOLO only

**Fig. 2:**
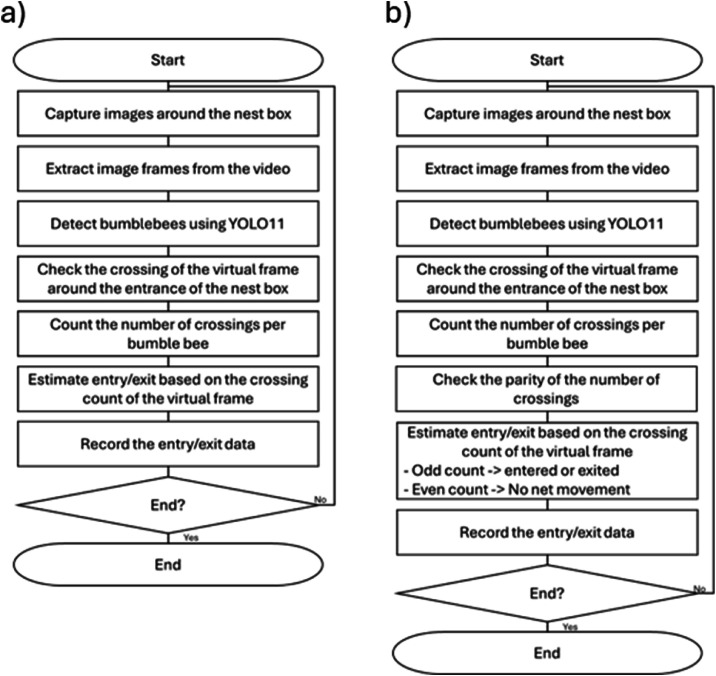
Flowchart of the detection methods using a) YOLO only and b) YOLO with a simple algorithm.

A flowchart of the YOLO-only method is shown in Fig. 2a. The number of bumblebees that entered or exited the virtual frame was counted from the video image captured on 11 February 2025 using YOLO. The number of bumblebees that entered or exited the virtual frame was considered as the number of bumblebees that entered or exited the nest box. Results obtained using YOLO alone were compared with those obtained visually to construct a confusion matrix.

2) YOLO with a simple algorithm

**Fig. 3:**
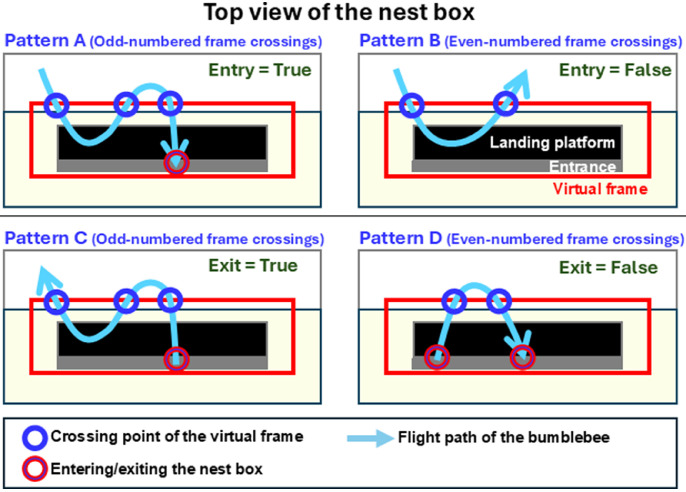
Criteria for determining nest box entry and exit, based on top-view images. Pattern A) entry into the nest box via odd-numbered frame crossings, Pattern B) no entry into the nest box via even-numbered frame crossings, Pattern C) exit from the nest box via odd-numbered frame crossings, and Pattern D) no exit from the nest box via even-numbered frame crossings.

A flowchart of the YOLO with a simple algorithm method is shown in Fig. 2b. In this method, we examined the same video image as that employed in the YOLO-only method, but with the addition of a simple algorithm. In this method, the number of times a bumblebee entered or exited the virtual frame (tracked using a unique ID) was evaluated. If the count was odd, the bumblebee was considered to have entered or exited the nest box (Fig. 3, Patterns A and C). However, if the count was even, the bumblebee was assumed to have turned back or returned to the nest box without fully exiting (Fig. 3, Patterns B and D). To distinguish bees that briefly entered and then exited the virtual frame (vice versa), a threshold was introduced for the time interval between two line-crossing points with the same track ID. Based on preliminary observations in this experiment, this threshold was set to less than one second. In the YOLO with a simple algorithm method, the process was performed after counting the number of bumblebees that entered or exited the virtual frame. Similar to the YOLO-only method, the numbers counted using YOLO with a simple algorithm were compared with those obtained visually to generate a confusion matrix.

### Performance metrics

From the confusion matrices obtained based on the comparison between the results of our prediction and visual observations in both methods, the performance metrics, including accuracy, precision, recall, and F1 score, were calculated using the following equations:

$$Accuracy=\frac{TP+TN}{TP+FP+FN+TN}$$

$$Precision=\frac{TP}{TP+FP}$$

$$Recall=\frac{TP}{TP+FN}$$

$$F1=\frac{2\cdot TP}{2\cdot TP+FP+FN}$$

## Method validation

**Fig. 4:**
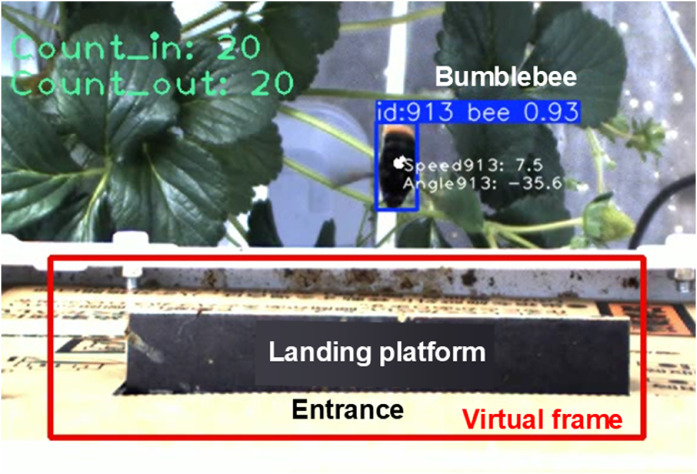
Detection of bumblebees around the nest box using YOLO.

Bumblebees were detected around the nest box using the YOLO model, as shown in Fig. 4. When using the YOLO-only method, bumblebees that entered or exited the nest box were miscounted, and a high occurrence of false positives (FP) was observed. However, when the YOLO with the algorithm method was employed, the performance metrics were significantly improved owing to the reduction in FP (Table 1). Accuracy, precision, and F1 score were 1.7 to 2.4 times greater in the YOLO with the algorithm method than in the YOLO-only method. The performance metrics showed that the number of bumblebees entering or exiting the nest box could be estimated with high reliability using YOLO with the algorithm method. This suggests that the proposed algorithm is effective in counting bumblebees entering or exiting the nest box.

**Table 1:** Performance metrics for a) YOLO only and b) YOLO with the algorithm.

	Performance metrics (%)	
	YOLO only	YOLO with the algorithm
Accuracy	41.3	95.5
Precision	41.3	97.0
Recall	100.0	96.2
F1 score	58.5	96.6

When bumblebees exhibited straight flight paths, the performance of the YOLO-only method approached that of the YOLO with the algorithm method. However, the straight flight paths of bumblebees were rarely observed, and single or multiple turns often occurred around the virtual frame set in front of the nest box. Hence, the YOLO-only method was not sufficient to accurately count the number of bumblebees that entered or exited the nest box, emphasising the importance of our YOLO with the algorithm.

In the YOLO with the algorithm method, FP were observed because the threshold of the time interval between two line-crossing points was greater than one second for a few bumblebees with the same track IDs. Preliminary observation showed that a reduction in this threshold increased FP and an increase in the threshold increased false negatives (FN). Based on the preliminary observations, a one-second threshold was adopted for this experiment. Additionally, accidental changes in the track ID of the bumblebees also contributed to FP. FN occurred when a single-track ID was assigned to two different bumblebees that had entered and exited the frame, respectively. Improving the accuracy of track ID assignment is expected to reduce both FP and FN. Although higher performance metrics were obtained in the YOLO with the algorithm method compared to the YOLO-only method, further improvement can be achieved using an appropriate threshold and by accurately assigning track ID.

When the bumblebees entered or exited the virtual frame without entering or exiting the nest box, FP was observed in the YOLO-only method (e.g., passing the virtual frame without entering). Odd-numbered entries or exits greater than three in the virtual frame led to the occurrence of FP. Even-numbered entries or exits greater than two also led to FP, but no entries or exits occurred in the nest box. Although efforts were made to optimize the size and shape of the virtual frame, FP remained high, and performance metrics were consistently lower compared to those in the YOLO with the algorithm method (data not shown). In the YOLO-only method, true negatives (TN) and FN were not detected. Although accuracy and precision were consistent, and recall reached 100%, the F1 score indicated that the method was not reliable enough to accurately estimate the number of bumblebees entering or exiting the nest box. These findings indicate the challenge of accurately counting bumblebees using the YOLO-only method.

To capture the rapid movement of bumblebees, the frame rate in this experiment was set to 240 fps. However, to reduce computational costs, a lower frame rate may be considered in the future by incorporating object tracking to maintain accuracy. Additionally, different YOLO model variants (e.g., YOLOv11n, YOLOv11s, YOLOv11m, and YOLOv11l) should be explored to optimize performance and efficiency. However, the optimal frame rate and model configuration remain undetermined. Furthermore, although 1,000 training images were used in model development, it is unclear whether this quantity is sufficient. Future research should focus on identifying an optimal balance among frame rate, model configuration, and training dataset size to improve performance and reduce computational costs.

## Limitations

In this study, model training was conducted using images captured only during the daytime, when bumblebees could be clearly observed. This limits the generalizability of the results, as bumblebee activity in the early morning and late afternoon was not included. To comprehensively monitor bumblebee activity throughout the day, the use of infrared cameras with suitable lighting will be necessary. Nonetheless, the current YOLO with the algorithm method is applicable for counting the number of bumblebees entering or exiting a nest box under standard daytime conditions.

## CRediT author statement

**Katsumi Ohyama**: Conceptualization, Investigation, Supervision, Data curation, Methodology, Validation, Writing – original draft. **Hiroki Naito**: Data curation, Methodology, Writing – review & editing. **Norio Hirai**: Methodology, Validation, Writing – review & editing.

## Related research article

N/A

## For a published article

None

## Declaration of competing interest

The authors declare that they have no known competing financial interests or personal relationships that could have appeared to influence the work reported in this paper.

## Data Availability

Data will be made available on request.
